# Perceived vulnerability related to health in cancer post-treated adolescent in Iran: a content analysis

**DOI:** 10.1186/s12889-024-19404-x

**Published:** 2024-07-16

**Authors:** Masumeh Akbarbegloo, Mahnaz Sanaeefar, Mehrdad Karimi, Marzieh Hoseini

**Affiliations:** 1 Department of Nursing, Faculty of Nursing and Midwifery, Khoy University of Medical Sciences, Khoy, Iran; 2 Health in Emergencies and Disasters Department, Shahid Sadoughi University of Medical Sciences, Yazd, Iran; 3 Department of Biostatistics, Faculty of Public Health, Khoy University of Medical Sciences, Khoy, Iran; 4 Unit of Intervention and Implementation Research for Worker Health, Shahid Motahari Hospital, Urmia University of Medical Sciences, Urmia, Iran

**Keywords:** Perceived vulnerability, Adolescents, Post-treated cancer

## Abstract

**Background:**

The prevalence of cancer is increasing, which significantly impacts the health and various aspects of the lives of cancer-post-treated adolescents. Adolescents with cancer have many negative consequences, including increased vulnerability. Therefore, this study aimed to explore the perceived vulnerability of cancer-post-treated adolescents using a deep examination of experiences and perceptions of participants.

**Methods:**

This study was conducted on 18 participants, who were selected based on purposive sampling in 2023 using a qualitative method through a content analysis approach in Iran. A face-to-face and semi-structured individual interview was used to collect data. Inclusion criteria were children aged 11–19 years, with no history of diseases except cancer, at least one year passed since their last treatment and were aware of their disease. Inclusion criteria for other participants were Parents of childhood cancer survivors whose child is under 19 years old. Health care providers that had at least one year of experience working with and caring for pediatric cancer survivors. The research objective, participation principle, and interview recording were explained to the participants before the interview started. The criteria of credibility, dependability, confirmability and transferability were included to support the trustworthiness of data. The data was analyzed using the conventional qualitative content analysis method and MAXQDA10 software was used for data management.

**Results:**

The participants included 12 cancer-post-treated adolescents, two parents, two nurses, one doctor, and one cancer charity representative. The data analysis identified three categories: “Confusion in early adolescence”, “Psychological turmoil of disease”, and “Physical burden of the disease”. Finally, the latent content was formulated in to a “Perceived vulnerability” overarching theme.

**Conclusion:**

Identifying the patient’s perceptions and beliefs is one of the current health problems to improve the quality of life and facilitate the optimal transition from adolescence to adulthood and adult care. Health professionals have an opportunity to address factors that increase survivors’ sense of vulnerability to health problems by correcting knowledge gaps.

## Introduction

The childhood cancer survival rate has increased significantly in recent years due to advanced treatments [1]. The average five-year survival of children with various types of cancer has risen to about 80% in developed countries [2]. Studies in Iran have also indicated that the survival rate of 5-year Iranian children is 65% for leukemia, 74% for Hodgkin, and 86% for Wilms’ tumor [3]. According to the definition of the American Cancer Society, cancer post-treated people are diagnosed with cancer from the end of the initial treatment until the balance in life [4].

Post-treated cancer people are exposed to a wide range of symptoms as short- and long-term effects related to cancer and treatments, including physical problems, adverse quality of life, psychosis, sexual problems, difficulties in establishing social relationships, and financial concerns after completing the initial treatment [5]. They have poorer general health than people without a history of cancer [6]. In addition, more than half of these survivors have limitations in physical performance, and a third of them have limitations in daily life activities [7], along with fatigue even years after treatment [8]. Overall, 22% of post-treated people experience fatigue within the first year after completing treatment [9].

There are confusion and general concerns in cancer, such as confusion in interpersonal relationships, dependence, and non-dependence, problems in reaching the goal, confusion in body and sexual image, integrity, and existence. However, specific patient needs also arise due to different age periods [10]. Cancer post-treated adolescents have problems and care needs due to the consequences of the disease and are affected by the developmental stage. When people try to become independent and form a positive self-concept of themselves and their relationships, dreaming about an illness or its complications during this period can be particularly challenging. Autonomy, relationships with peers, future orientation, and uncertainty of recovery or non-recurrence of the disease complicate the situation [11].

A psychological approach widely used by people dealing with a disease or a life-threatening factor is based on regulating the patient’s behavior. According to this approach, when faced with a disease, people create a general image and a specific belief about the disease and its treatment in their minds, which is called disease perception. This understanding of the disease is effective in the behavior, the person’s adaptation to the disease, the management of the disease by the person, and the treatment of the disease. Studies on disease perception have shown that disease perception includes five dimensions, including recognition of symptoms, duration of the disease, cause of the disease, outcome of the disease, and its curability [12]. Disease perception is related to some of the results of the illness, including care behaviors and individual management, which can be attributed to the influence of the patient’s beliefs on his behavior. Such beliefs affect children’s treatment, health care, mental and physical health, and acceptance and continuation of their treatment [13]. In addition, using appropriate methods to deal with these challenges can be helpful by increasing the number of patients cared for at home. Identifying perceived injuries in survivors and intervening based on the identified injuries is one of the effective solutions [14].

Perceived vulnerability is strongly influenced by one’s beliefs, and refers to a person’s belief about how vulnerable they feel to a stressful situation or health problem [15]. According to the HBM the probability of adopting appropriate coping strategies and precautionary self-care behaviors of a person to prevent the negative consequences of stress and diseases depends on his beliefs in the level of perceived vulnerability to diseases and stressful situations [16]. The results determined that older children suffer more damage than younger children due to a greater understanding of the disease process and physical problems related to the disease and its treatment. Therefore, these children need special attention and interventions to reduce vulnerability [17].

### Aim

Considering the importance of examining the perception of vulnerability and its impact on the care and treatment of patients, and on the other hand, the lack of documentation in the field of children’s experience or understanding of vulnerability to the disease, as well as the existence of individual and cultural differences in the perception of the disease, therefore this study aimed to explore the perceived vulnerability of cancer-post-treated adolescents. The results can help nurses, doctors, and policymakers of health promotion programs identify the problems of cancer-post-treated adolescents, provide educational and preventive interventions to face and reduce the harm caused by the disease appropriately.

## Methods

### Study design

This qualitative study was conducted with a Conventional Qualitative Content Analysis approach to discover and understand the inner world of people. Since experiences form the structure of truth for each person, the researcher can only discover the meaning of phenomena from their point of view by entering the world of people’s experiences [18]. The present study investigated the perceived vulnerability of cancer-post-treated adolescents with this method and deeply examined the experiences and perceptions of the participants.

### Study sample

The participants were selected from children’s educational centers and the Omid Urmia and Khoy charity institutions using purposive sampling until data saturation. The inclusion criteria were children aged 11–19 years, with no history of other physical and mental diseases except cancer (through the patient’s medical record), and children who had at least one year passed since their last treatment and were aware of their disease. The exclusion criteria included lack of access to children at any study stage (mortality and recurrence of the disease) and lack of information about the disease. Because parents are involved in caring for adolescent cancer survivors, most of the adolescents interact with doctor, nurse, and charity staff during treatment and posttreatment of cancer, and they play a valuable role in providing support to survivors [11]; Therefore, to collect richer information, they were included as sub-participants based on the study inclusion criteria, and their data combined with the data obtained from interviews with adolescents. Inclusion criteria for sub-participants were: Parents of childhood cancer survivors whose child is under 19 years old. Health care providers that had at least one year of experience working with and caring for pediatric cancer survivors. Exclusion criteria were the presence of cancer or other chronic diseases.

Finally, the participants consist of 12 cancer-post-treated adolescents as a key participant, and two parents, two nurses, one doctor, and one cancer charity representative as a sub-participant (generally 18 participants). Interviews continued until data saturation, in which the collected data were repeated the previous data, and no new information was obtained by continuing to collect data [19]. In this study, data saturation was achieved up to the 16th interview, according to the research group, and two more interviews were conducted to ensure no new data and results.

### Data collection

A face-to-face interview was used to collect data by second researcher of study. The interviews started unstructured and gradually turned into semi-structured interviews with the emergence of classes to collect more comprehensive and complete information. The interview guide was used to ensure that all topics were covered. Each interview lasted approximately 40 to 55 min. The research objective, participation principle, and interview recording were explained to the participants before the interview, and the interview began after obtaining informed consent. The families did not impose restrictions on the children’s interviews or accompany them during the interviews, and the children were interviewed individually.

Second researcher of the study that was expertise and experience in qualitative studies conducted the interviews. At the beginning of each interview, participants’ demographic information, history of cancer, and how to find out about this disease were asked, and then the interview began with a general question. Thus, the participants were asked to *“talk about their experience of post-treatment problems and concerns?“*. Physicians, nurses, families, etc. were also asked: *“During the period after the treatment*,* what problems and concerns did the children face?”* or *“Tell me about your experience”*. Based on the answers and data analysis, explorative interview questions such as “*Tell me more about this*” and “*What does this mean*” were asked. At the end of all the interviews, some more open questions were asked, such as “*Do you think there is anything you didn’t say*?” All interviews were recorded by audio recorder. Each participant was interviewed once. The data collection lasted almost five months, and the entire research was conducted between July 2022 and July 2023 for 12 months.

### Setting

Cancer-post-treated adolescents have a treatment follow-up plan and go to the doctor’s or hospital clinic. Therefore, the interviews were conducted face-to-face with prior arrangements by the children and their families in the researched hospitals in Urmia and Khoy (the interview location is the room or the hospital chapel). Charity organizations helped some cancer-post-treated adolescents in Urmia and Khoy. Therefore, an employee of the Cancer Charity Association was interviewed at their workplace. The interviews with the nurses were also conducted in nursing rest room, and the interviews with the doctors were conducted in their office with a prior appointment. During the interview, it was tried to observe the standards of physical space, such as not going to and from the interview room, closing the door and window of the room to prevent noise pollution and keeping the light and temperature of the room balanced.

### Data analysis

**Fig. 1:**
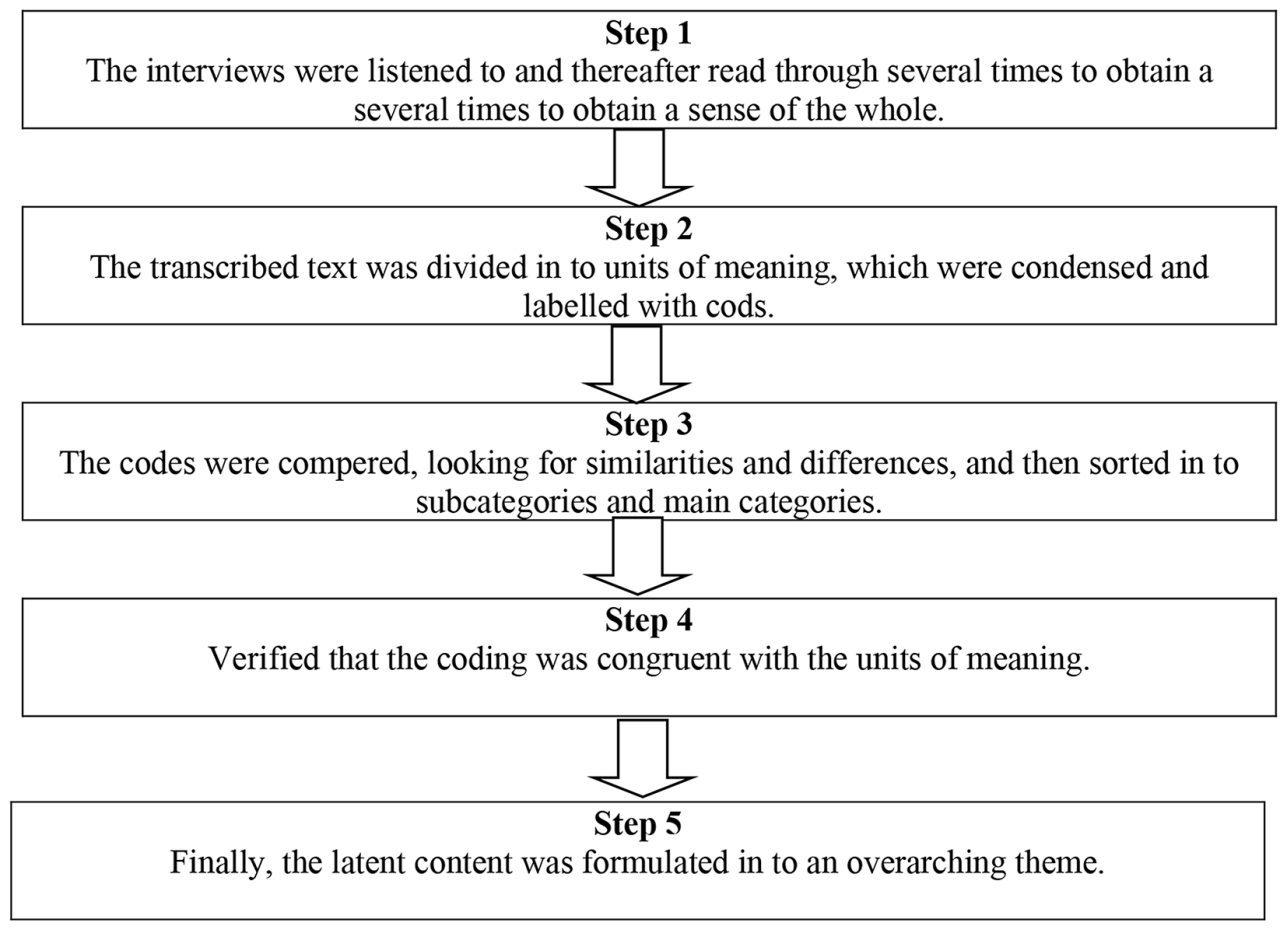
Five steps of doing qualitative content analysis. Figure adapted from Graneheim and Lundman (2004)

The data were analyzed using the Conventional Content Analysis method. Before starting the data analysis, the researcher does not use the existing theory or previous studies to create the initial codes and search for the data’s hidden meaning. As the stages of analysis progress, initial code design, revision, and refinement are carried out [18]. The steps proposed by Graneheim and Lundman (Fig. 1) were used to perform qualitative-contractual content analysis [20]. Therefore, in the first step, the text of the interviews was implemented word by word on paper and used as the primary data of the research. In the second step, the text was divided into semantic units that were summarized and shortened. In the third step, the abstraction of semantic units and selection of codes was designed. According to the participants’ experiences, overt and hidden concepts were determined as sentences or paragraphs of their words and denoting codes, and then coding and purification were performed. In the fourth step, the codes that denoted a single topic were placed in a class based on the continuous comparison of similarities, differences, and fit and categorized into subclasses and classes. Ambiguous points that needed attention were investigated by the research team and participants so that the ambiguities were resolved and the location of the codes on each floor was entirely determined. This process was continued by one researcher (corresponding author) as far as was reasonable and possible, and MAXQDA10 software was used for data management.

### Trustworthiness

The main task of a researcher is to validate the data to ensure the correctness of the data [21]. Lincoln and Goba’s (Table 1) suggested methods were used to ensure the accuracy and robustness of the study data [22]. The credibility of data was verified by leaving aside the thoughts and assumptions, recording the research process, documenting, not being biased, prolonged engagement and refraining from an in-depth review of the texts, checking the data with samples and experts. Participants’ survey was used informally during the research to ensure the confirmability of the data. By transcribing the text as soon as possible (on the same day of the interview) and making it available to some participants, they were asked to read the manuscripts and check the accuracy of their similarity with their expressed experiences. This process was conducted by establishing good communication with the participants and gaining their trust in the researcher. For dependability of findings two external observers with a Ph.D. in nursing with experience in qualitative research were asked to review the interviews and the initial coding and categories for the consistency of the coding as the stability and reliability of the data in similar times and conditions [23]. In this study agreement between two supervisors calculated by using Holsti’s coefficient of reliability (score 7 ≥ is acceptable) [24]. For example, the level of agreement with 25 codes (14 codes for the researcher, 11 codes for other researchers, and ten standard codes) is equal to 0.8. Therefore, coding method seems appropriate based on the minimum agreement coefficient. The cases with disagreements were identified in this revision and discussed and exchanged opinions about them in a meeting. Finally, the closest code or category was selected with the agreement of the research team. The research details, including the study background and the characteristics and experiences of the participants, were well described for data transferability, in addition, purposive sampling with maximum diversity helps data transferability.

**Table 1:** Lincoln & Guba’s (1985) trustworthiness criteria & techniques for establishing them

Criteria	Definition	Techniques
Credibility (internal validity)	Confidence in the ‘truth’ of the findings	1) Prolonged engagement
		2) Persistent observation
		3) Triangulation (sources, methods, investigators)
		4) Peer debriefing
		5) Negative case analysis
		6) Referential adequacy (archiving of data)
		7) Member checks
Transferability (external validity)	Showing that the findings have applicablity in other contexts	8) Thick description
Dependability (reliability)	Showing that the findings are consistent and could be repeated	9) Overlap methods (Triangulation of methods)
		10) Dependability audit- examining the process of the inquiry (how data was collected; how data was kept; accuracy of data)
Confirmability (objectivity)	A degree of neutrality or the extent to which the findings of a study are shaped by the respondents and not researcher bias, motivation, or interest	11) Confirmability audit- examines the product to attest that the findings, interpretations & recommendations are supported by data

### Findings

The participants included 12 cancer-post-treated adolescents, two parents, two nurses, one doctor, and one cancer charity representative. The average age of adolescents was 15.14 ± 2.87 years, the average age of diagnosis was 8.14 ± 4.16 years, and the average duration of treatment discontinuation was 4.39 ± 3.18 years (Table 2).

**Table 2:** Participant demographic information based on data from childhood cancer survivors

Participants	Diagnosis	Duration of treatment interruption*	Age of disease diagnosis*	Current age of adolescents*
Adolescent	ALL	3	6	12
Adolescent	ALL	1	13	16
Adolescent	Osteosarcoma	1.5	10	14
Adolescent	Ewing sarcoma	6	8	19
Adolescent	Hodgkin’s lymphoma	1	15	17
Adolescent	ALL	4	14	19
Adolescent	Hodgkin’s lymphoma	1	10	14
Adolescent	ALL	3	5	12
Adolescent	Wilms Tumor	4	5	11
Father	Osteosarcoma	3	12	17
Mother	Medulloblastoma	6	3	12
Adolescent	ALL	11	4	18
Adolescent	Retinoblastoma	8	3	13
Adolescent	Rhabdomyosarcoma	9	6	18
(Mean ± SD)	-	4.39 ± 3.18	8.14 ± 4.16	15.14 ± 2.87

*Age presented is in years

The data analysis on the perceived vulnerability in cancer-post-treated adolescents resulted in categories, including “Confusion in early adolescence”, “mental turmoil of the disease” and “physical burden of the disease”. These three categories defined a main category or common theme under the title “perceived vulnerability” (Table 3). Each of the categories and subcategories is explained below.

**Table 3:** Results of qualitative data analysis

Subcategory	Category	Main category
Threat of independence	Confusion in early adolescence	Perceived vulnerability
School problems		
The feeling of being rejected by friends and family		
Uncertainty about the occupational future		
Emotion fluctuation	Psychological turmoil of disease	
Doubts about recovery		
Deterioration of the mental image		
Fear of cancer labeling		
Remaining physical effects of the disease	The physical burden of disease	
Challenges of sexual maturity and fertility		

### Confusion in early adolescence

Confusion during adolescence was one of the things that the participants mentioned. Adolescence is the stage of fundamental changes in different physical, psychological, and social dimensions, which create new needs. Along with the crisis and transformation adolescents experience in this age period, suffering from chronic diseases causes particular problems and needs. Therefore, adolescence cannot control their past and future, which cause them to remain confused. This category has four subcategories: “threat of independence,” “school problems,” “feeling left out from friends and family,” and “uncertainty about a future career.”

- Threat of independence.

Some adolescents were bothered by dependence on others in routine and daily life tasks and did not want to burden others. Affected adolescents try to do their work by themselves as much as they can, deny any dependence on others, and even desire to do the job and follow up treatment procedures alone. One of the adolescents said in this regard:

*As much as possible*,* my pride does not allow me to do my work for others; even when my foot hurts*,* I sit and go forward*,* and I don’t want to tell my family to come and hold my hand*,* to get up* (P5)

- School problems.

The participants believed that absenteeism from school occurs for various reasons, such as tiredness and long-term intolerance of the classroom, illness follow-up, and catching infectious diseases after the teenager returns to school. On the other hand, some teenagers have weakness and slow learning speed due to the complications of the disease, which cause a drop in education, lagging in education, and academic problems in adolescents. According to one of the adolescents:

*I used to do my homework*,* but during those few months when my lungs were infected and I had a cough and chest pain*,* I couldn’t go to school anymore*,* and I studied at home* (P5)

Or another adolescent said about dropping behind in education:

*I dropped behind my studies due to my illness*,* and when I returned to school after treatment*,* they said that I should study at an adult school. If possible*,* I should study some lessons in my school to introduce myself to my classmates. Otherwise*,* if I continue like this*,* I won’t be able to participate in the future entrance exam with the rest of my classmates* (P7)

The mother of one of the adolescents said about the effect of radiation therapy on her child’s brain:

*My daughter*,* at the age of 11*,* who was in the fifth grade*,* underwent chemotherapy and radiation therapy*,* but unfortunately*,* after that*,* she suffered a severe drop in education. The teachers at school said it is better for me to take my daughter to a school for mentally retarded children and study with them so that she is not bothered* (P12)

- The feeling of being rejected by friends and family.

Some cancer-post-treated adolescents reduced their communication with their family and surrounding people due to the fear of worsening the disease, which caused them to feel lonely, rejected, and separated from their friends and family. After returning to school, some adolescents did not know how to deal with their classmates and explain their disease or the reason for their absence. Therefore, cancer-post-treated adolescents felt withdrawn from their old friends and lost the acceptance of their friends. One of the adolescents saved from cancer said:

*My parents are always afraid that if I am with my friends or go to our family’s house*,* my disease will get worse or I will get an infection. That’s why my communication with friends and family has decreased* (P2)

The mother of one of the cancer-post-treated adolescents stated:

*Dealing with these children at school is a little different. One day when I went to school*,* I saw my daughter holding her hands and standing in the corner of the school yard. I said why don’t you go play sports with the children? She said Mom*,* the children say that you are sick*,* and if you fall and bleed*,* they will say that we did this* (P12)

- Uncertainty about the occupational future.

The families of most of the participants in this study had financial problems. Therefore, financial independence was one of the things that adolescents mentioned at an older age. On the other hand, the participants believed that they could not work in some normal jobs in society due to the complications of the disease, and they were worried about finding a suitable job with physical strength in the future. They preferred to enter jobs that do not require heavy physical work and much movement, and one of the adolescents expressed:

*Maybe my father will not have a good financial situation in the future to be able to cover all the expenses of my illness. For example*,* I am thinking of learning computer because it does not require high physical strength*,* maybe it will help me*,* and I can work in an institution or other places in the future and become independent* (P16)

### Psychological turmoil of disease

Most cancer-post-treated adolescents stated that fears and worries persist even after treatment and recovery. The possibility of disease recurrence was the biggest concern, and most participants believed that stress during the disease period and after it caused the failure of the treatment plan. On the other hand, long-term treatments before the treatment discontinuation period have caused mood changes. In addition, the effects of the disease on the person’s appearance damaged the mental image of the body. Some post-treated patients and their families hid the disease from their friends and family due to the fear of the cancer taboo. This category had four subcategories of “emotions fluctuating”, “doubts about recovery”, “deterioration of the mental image”, and “Fear of cancer labeling”.

- Emotion fluctuation.

Post-treated adolescents who experienced the chemotherapy period believed that treatment has many psychological and physical side effects. Before stopping the treatment, long-term treatments have caused bad moods, quick anger, lack of positive outlook in the post-treatment period, and discomfort in remembering the disease in them. Some adolescents also had dual feelings after stopping the treatment, one of which was due to happiness at the end of the treatment period, and the other was due to the fear of the return of the disease. One of the adolescents said about the bad mood caused by the disease and treatment:

*In general*,* I was very depressed during the treatment*,* I was nervous*,* and I was not in the mood for anything*,* but when I finished the treatment*,* I got a little better*,* although I still get angry sometimes* (P8)

The mother of one of the adolescents said about the mental pressure caused by the remainder of the illness by her child:

*As for his/her feelings*,* he/she doesn’t like to talk about his disease too much. He/she says I am bothered and don’t like it. Sometimes we talk or ask him/her about this*,* but he/she gets upset and says don’t talk about it at all and gets upset. In my opinion*,* they have gone through a tough time*,* and they were in the hospital for treatment for a year. His medications were heavy*,* and he was given platelets and blood regularly. That’s why he/she says that everything is a memory for me and I think about that day*,* and he says that he/she doesn’t like to talk about that day or his/her disease* (P12)

- Doubts about recovery.

Most of the participants were afraid of getting sick again and were uncertain about the treatment of their disease. There was concern about the result of treatment and tests during the follow-up period or going to the doctor or hospital in case of physical symptoms and the emergence of a specific problem. One of the post-treated adolescents said

*Anyway*,* medical futility is a perfect day. I had so much fun that I don’t vomit anymore. I don’t need to go to the hospital and get a vein. I was pleased when they told me that it was my last session and that my treatment was stopped for the time being. After that*,* another thing will start*,* lest I go to take a test now*,* they will say that the markers are high. Don’t let it metastasize now that I have bone pain. You get very stressed when they tell you to go for a test or a diagnostic work to get an answer. I’m not saying that cancer will kill you*,* but the suffering you have to go through is excruciating for you. It means chemotherapy again*,* nausea again*,* mouth sores again*,* and those problems start again. You are worried about it and always have doubts as if you don’t have peace* (P5)

- Deterioration of the mental image.

The participants believed that if the effects of the disease harm the attractiveness of the teenager and are visible in terms of appearance, it will damage the mental image of the body. The father of one of the adolescents expressed in this regard:

*Whenever my son wants to go to his friend*,* he is a little upset. He is afraid of being ridiculed in front of his friend because his leg is like this* (P13)

- Fear of cancer labeling.

Except for a few cases, most post-treated people and their families hid the disease from their friends and family. Only the first-degree family of the teenager knew about the disease because they were afraid of being labeled with cancer and believed that the name of the disease and chemotherapy was terrible and that there was no culture in the society. One of the adolescents stated:

*Unfortunately*,* chemotherapy or cancer has a bad name; it is neither cultured nor fully informed. When someone hears that someone has cancer*,* for example*,* you see that they quickly slap him on the back in front of his eyes*,* which means that he will not get the disease. There are other superstitions*,* but in any case*,* this is a big blow for us; what do I have that immediately hit him? Cancer is now in every family*,* but we must make our vision positive. None of my friends at school know about my illness even after stopping the treatment because of these issues*,* and we only told my teachers* (M18)

### The physical burden of disease

Based on the participants’ experiences, the physical burden of the disease was one of the influential factors in the self-care of cancer post-treated. Cancer-post-treated adolescents faced many physical and long-term complications during the treatment period, which affected the regular life routine of these people. There are two categories of “remaining physical effects of the disease” and “challenges of sexual maturity and fertility” in this category.

- Remaining physical effects of the disease.

Participants mentioned the impact of cancer on various systems of the body. There were many physical complications such as kidney failure, the need for dialysis, involvement of the nervous system, blindness or numbness of the limbs, movement problems in the legs, various infections in the body, especially susceptibility to influenza, and sexual system problems such as menstrual disorders. One of the adolescents’ fathers stated about the nervous system’s complications:

*When I look at my daughter with children of her age*,* she is behind in some things; for example*,* walking or running. She does these things*,* but her movements are slow. She cannot find things when she falls on the floor. She sees big things but has trouble seeing small things; for example*,* she can’t find a cleaning cloth that falls on the floor. I see her dragging her hand on the floor to find it* (P14)

One of the adolescents said about the complications of the kidney system:

*During the treatment*,* the doctor told me that I also had a kidney problem. At first*,* he gave me medicine at first*,* but then he said I should undergo dialysis. After medical futility*,* we went to Tehran with my parents to solve the problem. But they said that my kidneys are not working well and dialysis is the best solution for now*,* so we can see what happens later* (P16)

- Challenges of sexual maturity and fertility.

The participants were worried about puberty, such as period disorders and the possibility of infertility in the future, especially in females. On the other hand, they were concerned about notifying the other party about the disease during marriage or transferring it to their children. One of the adolescents expressed about the period disorder:

*During the treatment of the disease*,* I took LD medicine. After medical futility*,* my doctor said I don’t need to use it anymore*,* but I am 17 years old and still haven’t had my period. I talked to my doctor about this*,* and he says that it’s not a problem*,* it’s just that you might have a late period*,* but I and my family are apprehensive about it* (P7)

## Discussion

Creating and maintain healthy habits is important for everyone; It is especially important for cancer survivors. cancer-post-treated adolescents have to resume lives profoundly transformed by the cancer experience. Therefore, the transition from the end of treatment to post-treatment is associated with a new set of challenges for cancer-post-treated adolescents that usually limited attention has been paid to this critical reentry period [25].

Confusion in early adolescence was one of the categories obtained in the present study. Conway Keller et al. (2020) also reported that cancer-post-treated adolescents feel confused, unprepared, and lonely [26]. There are four subcategories of school problems, threats to independence, uncertainty about the future of employment, and the feeling of being excluded from friends and family for this category. Otth and Scheinemann (2022) showed that children with cancer are prone to various cognitive or learning problems. This cognitive impairment is usually seen a few years after the child has endured cancer treatment. When cancer-post-treated children return to school, they may experience poor test scores, memory, attention, behavior problems, poor hand-eye coordination, and development over time. If these children’s cognitive learning disabilities affect their educational success, parents must request special education services for their children at school [27]. Walker et al. (2019) also found that some adolescents mention “chemo brain” as a side effect of their treatment, which may be related to brain damage caused by brain tumors or central nervous system metastasis or side effects of cancer treatments such as chemotherapy and radiation therapy. Chemotherapy and radiation treatments may damage the brain’s white matter and disrupt brain activity [28]. This cognitive problem is known as post-chemotherapy cognitive impairment or “chemical brain,” commonly used by cancer post-treated with thinking and memory problems after cancer treatment [29].

The probability of health-related unemployment in post-treated childhood cancer between the ages of 30 and 50 is higher than in other populations; cancer affects post-treated employment status more than long-term income [30]. Therefore, uncertainty about the job future was one of the concerns of the post-treated in the present study. A cohort study of almost 100 post-treated groups worldwide proved that the level of work ability among the post-treated appeared during the first two years of a cancer diagnosis. About two-thirds of post-treated childhood cancer have been employed, and the rest have been unable to work due to complications of the disease [31]. On the other hand, Janssen et al. (2022) showed that childhood cancer survivors are often unemployed [25]. Therefore, comprehensive survival care and interventions should be provided to post-treated adolescent at risk of health-related unemployment to obtain and maintain suitable employment status.

The threat of teenage independence was another subcategory. Brinkman et al. (2018) also determined that 60% of post-treated children with central nervous system (CNS) tumors do not achieve complete independence in adulthood [32]. Larsen et al. (2022) indicated that cancer in adolescence leads to limitations in daily activities, disability, and fatigue of the adolescent and disrupts independence, which requires management and care [33].

The feeling of being rejected by friends and family was one of the subclasses of the present study. Interaction with peers has been described as essential in helping to develop a sense of identity and independence threatened by illness in adolescents with cancer [34]. According to estimates, 11% of children with cancer suffer from school phobia. Affected adolescents’ absence from school is often due to medical treatment or disease conditions, which can be related to fear of rejection by classmates or fear of overprotective parents [35]. Hotchkiss et al. (2023) examined experiences and attitudes related to peer interactions among post-treated cancer adolescents and young adults. The results showed that rejection by peers and reduction of interactions due to deficiencies in social skills of adolescents with chronic diseases lead to incompatibilities and psycho-social inefficiencies in adulthood [36]. McDonnell et al. (2018) also revealed that children with weak relationships with their peers were at a lower level in academic progress, social adequacy, identity acquisition, and general adjustment [37]. Family and peer support is essential and shows the need for interventions such as camps to help adolescents and young adults develop and maintain support networks during treatment and post-treatment [38].

The psychological turmoil of disease was one of the category with four subcategories, such as emotion fluctuation. Studies by researchers at the University of Maryland in the United States of America showed that people who were diagnosed with cancer and were treated during adolescence are very vulnerable and have significant differences from others psychologically. This study examined 523 cancer survivors aged 15 to 39 and showed that these people have less desire to continue their education, get married, become independent, and have full-time jobs compared to their siblings and other people. Further, cancer post-treated people are twice as likely to suffer from depression, mood disorders, and anxiety compared to their siblings, and these people need help from their families imperceptibly [39]. Studies have indicated that parents are less inclined to mention their children’s diseases or problems after stopping their chemotherapy. For this reason, parents’ reports on children’s quality of life may be affected by bias recall [11]. Experts believe that special social measures should be taken for these people to continue their activities with the maximum efficiency of their mental and physical abilities. Therefore, getting advice from a social worker who is acceptable to these people can be helpful.

Doubt about recovery was another subcategory of the present study. Vandraas et al. (2021) concluded that fear of disease recurrence was a frequent concern among survivors of young adult cancers more than 15 years after diagnosis, and 74% of them worried that they would have another type of cancer [40]. Yang et al. (2019) emphasized that psychological distress and more severe and extensive treatment were associated with higher fear of disease recurrence. Lower scores on levels of physical functioning, mental health, and overall health-related quality of life (QoL) were identified as consequences of doubting recovery [41]. Age-appropriate and flexible psychological care would be more successful potentially with this crucial background information.

Deterioration of mental image was one of the subcategories that obtained. In Brierley et al. (2019), participants described the effects of treatment on physical appearance, such as hair loss, scarring, and weight gain, which led to body dissatisfaction, shame, and decreased sexual health [42]. This happens when people may be more aware of their bodies and start new intimate relationships. Few people with body image concerns seek help and support [43]. Cancer-post-treated adolescents may benefit from family and peer support, healthy lifestyle interventions, and clinical environments that cater to appearance sensitivities.

Fear of the cancer label was one of the subcategory of present study. According to the culture of Iran and the lifestyle of Iranian families, Iranian people often interact with their first and second-degree families and relatives. For this reason, all parents stated that a family member or their family-in-law labeled them at least once. Perez et al. (2020) showed that being labeled is one of the problems of cancer post-treated people, which makes them less likely to be present in public gatherings and places to avoid being looked at with pity. In addition to the labeled people, those around him are also affected [44]. Since cancer has been considered an inevitable cause of death for many years, false beliefs about it are not strange.

AYA post-treated of cancer people report more medical and physical conditions, health-related disability and functional limitations. Therefore, the physical burden of the disease was one of the category, which had two subcategories, including residual physical effects of the disease and challenges of sexual maturity and fertility. Studies have also revealed that more than half of children undergoing cancer treatment develop some form of cardiac abnormality [45] and are at risk of developing adverse effects on the kidneys and liver. The risk of late liver side effects s in childhood cancer survivors is increased in those who have undergone liver radiotherapy and in those with factors such as higher body mass index and chronic viral hepatitis [46]. Many children and adolescents treated or undergoing cancer treatment often experience severe physical and cognitive effects caused by the treatment during the treatment process, which remain stable even after the completion. The presence of neuropsychological defects and problems in cognitive executive function is one of the side effects that has been the focus of many studies today [47], compromising the advantage of increased survival. Therefore, providing supportive care after stopping the treatment helps to return to normal life in adolescents.

The most physical concerns reported in the current study were hormonal and reproductive concerns, and therefore sexual maturity and reproductive challenges were among the subcategories obtained. In recent studies, about 51% of female and 44% of male survivors of childhood cancer reported fertility concerns [48, 49]. This finding is consistent with that of Levine et al., who showed that 49% of adolescents reported negative effects on sexual function after stopping treatment up to two years after treatment [50]. Most respondents in the current study who reported concerns about sexual performance did not seek help. These results indicate the need for regular screening to assess sexual function in cancer post-treated people.

### Strengths and limitations

This study has limitations that may limit the application of the findings; One of the limitations of the present study was that in order to observe accuracy in the study and minimize disturbing factors, the study was conducted in the form of individual interviews with children, which increased the implementation process and was time consuming. Like all qualitative studies, the generalizability of the results is limited, and it is necessary to repeat the study in different cultural conditions. Another limitation of the present study was the need for easy access to the research samples due to the high dispersion in the cities of West Azerbaijan province. In order to solve this restriction, in addition to Khoy, Urmia city was tried to be used as a centralized city where post-treated cancer children come from different cities of the province for follow-up treatment. One of the strengths of the current study was greater contact of the researcher with the children and their families in different spaces of the hospital, clinic and charity institutions, as well as the increase in the duration of the interviews as long as the participants wanted to continue, were the strengths of the present study that these factors played an effective role in clarifying the mental content of children and their families.

### Clinical implications/future direction

General conclusions related to the vulnerability of post-treated cancer adolescents due to the limited number of available qualitative studies are limited. Survey tools, such as the Patient Reported Outcomes Measurement Information System and a system of measures developed to collect patient-reported health status for physical, mental, and social well-being, can be used for further research in this vital area. Coordinated actions provide a better opportunity to understand the challenges of different cancers and their treatments. The results can be helpful and practical for health-treatment workers toward correct patient encounters. The results can also be used by researchers, managers, and planners to better understand the needs of cancer survivors and for proper and evidence-based planning.

## Conclusion

Our data highlighted that childhood cancer survivors and even their families remain vulnerable years after cancer treatment is completed. They confirm less adaptive health beliefs and are more endangered. Improving the provision of information about late effects to children and families may improve their engagement with survivorship care and monitoring.

Based on the results obtained from the study, childhood cancer survivors are facing academic and job problems that caused confusion in early adolescence. Identifying childhood cancer survivors who are at risk for adverse educational, occupational, and financial outcomes is important for developing appropriate support strategies for cancer patients and survivors throughout their cancer journey. For the successful return of survivors to school or workplace, Iran’s health system uses the support of key people such as peers, liaison teachers at school and home to improve educational activities, as well as employers, government organizations and NGOs to provide financial assistance.

On the other hand, identifying the patient’s perceptions and beliefs about physical and mental health problems effectively improves the quality of life and facilitates the optimal transition from adolescence to adulthood and adult care. Iran’s health system encourages behaviors that promote physical and mental health in survivors through providing personalized health counseling and correcting knowledge gaps by experts, that addressing these factors improves and influences behavioral practices.

## Data Availability

The datasets generated and analyzed during the current study are not publicly available due to the Iranian ethical review regulation. Data are available upon reasonable request. Inquiries for data access should be sent to Nursing Department, Khoy University of Medical Sciences, Iran, or contact the principal investigator Masumeh Akbarbegloo, m_Akbarbegloo@khoyums.ac.ir.

## Abbreviations

HBM: Health belief model
AYA: Adolescent and young adult
ALL: Acute lymphoid leukemia

## References

[1] Atun R, Bhakta N, Denburg A, Frazier AL, Friedrich P, Gupta S, Lam CG, Ward ZJ, Yeh JM, Allemani C, Coleman MP, Di Carlo V, Loucaides E, Fitchett E, Girardi F, Horton SE, Bray F, Steliarova-Foucher E, Sullivan R, Aitken JF, Banavali S, Binagwaho A, Alcasabas P, Antillon F, Arora RS, Barr RD, Bouffet E, Challinor J, Fuentes-Alabi S, Gross T, Hagander L, Hoffman RI, Herrera C, Kutluk T, Marcus KJ, Moreira C, Pritchard-Jones K, Ramirez O, Renner L, Robison LL, Shalkow J, Sung L, Yeoh A, Rodriguez-Galindo C (2020). Sustainable care for children with cancer: a Lancet Oncology Commission. Lancet Oncol.

[2] Miller KD, Siegel RL, Lin CC, Mariotto AB, Kramer JL, Rowland JH, Stein KD, Alteri R, Jemal A (2016). Cancer Treatment and Survivorship statistics. CA CANCER J CLIN.

[3] Hassanipour S, Fathalipour M, Delam H, Ghorbani M, Abdzadeh E, Arab-Zozani M, Riahi S, Salehiniya H (2019). The incidence of Childhood Cancer in Iran: a systematic review and meta-analysis. Iran J Ped Hematol Oncol.

[4] American cancer society. Cancer facts and Fig. 2018. Atlanta: American cancer society; 2019.

[5] Prasad M, Goswami S. Barriers to long-term follow-up in adolescent and young adult survivors of childhood cancer: Perspectives from a low-middle income setting. Pediatr Blood Cancer. 2021; 68 (12): e29248, 2021. 10.1002/pbc.29248. 10.1002/pbc.29248 34286916

[6] Malhotra P, Kapoor G, Jain S, Jain S, Sharma A (2021). Obesity and Sarcopenia in survivors of Childhood Acute Lymphoblastic Leukemia. Indian Pediatr.

[7] Prasad M, Chinnaswamy G, Vora T, Goswami S (2019). Burden of late effects and challenges faced in the long-term follow-up of paediatric germ cell tumour survivors: a report from India. Eur Urol Suppl.

[8] Valizadeh L, Zamanzadeh V, Ghahramanian A, Musavi S, Akbarbegloo M, Chou FY (2020). Adolescent cancer survivors’ experiences of supportive care needs: a qualitative content analysis. Nurs Health Sci.

[9] Prasad Maya S, Goswami G, Chinnaswamy SD, Banavali (2022). Long-term outcomes in survivors of Childhood Cancer: a 30-Year experience from India Kurkure. JCO Glob Oncol.

[10] Akbarbegloo M, Zamanzadeh V, Ghahramanian A, Valizadeh L, Matin H (2020). Health and Psychosocial Self-Care needs in off-therapy Childhood Cancer: Hybrid Model Concept Analysis. Patient Prefer Adherence.

[11] Neves MC, Bártolo A, Prins JB, Sales CMD, Monteiro S (2023). Taking care of an adolescent and young adult Cancer survivor: a systematic review of the impact of Cancer on Family caregivers. Int J Environ Res Public Health.

[12] Kern de Castro E, Benicio Beltrão G, Armiliato MJ, Peloso F, Gregianin LJ (2022). Illness perceptions in childhood cancer survivor and caregivers’ dyads. Clin Child Psychol Psychiatry.

[13] van Erp LME, Maurice-Stam H, Kremer LCM, Tissing WJE, van der Pal HJH, de Vries ACH, van den Heuvel-Eibrink MM, van der Heiden-van der Versluys BAB, Huizinga GA, Grootenhuis MA (2021). A vulnerable age group: the impact of cancer on the psychosocial well-being of young adult childhood cancer survivors. Support Care Cancer.

[14] Toruner EK, Altay N (2018). New trends and recent care approaches in Pediatric Oncology nursing. Asia Pac J Oncol Nurs.

[15] Zanatta EA, Silva KF, Silva CB, Brum MLB, Motta MGC (2021). Children and adolescents with cancer: vulnerabilities and implications on the right to health. Rev Gaúcha Enferm.

[16] Miller LH, Smith AD (1987). Vulnerability to stress scale.

[17] Khadra C, Le May S, Tremblay I, Dupuis F, Cara C, Mercier G, Vachon MF, Lachance Fiola J (2015). Development of the adolescent Cancer suffering scale. Pain Res Manag.

[18] Graneheim UH, Lindgren BM, Lundman B (2017). Methodological challenges in qualitative content analysis: a discussion paper. Nurse Educ Today.

[19] Monique H, Kaiser BN (2022). Sample sizes for saturation in qualitative research: a systematic review of empirical tests. Soc Sci Med.

[20] Graneheim UH, Lundman B (2004). Qualitative content analysis in nursing research: concepts, procedures and measures to achieve trustworthiness. Nurse Educ Today.

[21] Cloutier C, Ravasi D (2021). Using tables to enhance trustworthiness in qualitative research. Strateg Organ.

[22] Lincoln YS, Guba EG (1985). Naturalistic Inquiry.

[23] O’Connor C, Joffe H (2020). Intercoder Reliability in Qualitative Research: debates and practical guidelines. Int J Qual Methods.

[24] Holsti OR (1969). Content analysis for the social sciences and humanities.

[25] Janssen SHM, van der Graaf WTA, van der Meer DJ, Manten-Horst E, Husson O. Adolescent and young adult (AYA) Cancer Survivorship practices: an overview. Cancers (Basel). 2021 28; 13(19):4847. 10.3390/cancers13194847. 10.3390/cancers13194847 PMC8508173 34638332

[26] Conway Keller M, King C, Hart L, Engelke K, Needham A, Holden E, Foy K, Lucas R (2020). The end of cancer treatment experience for children, adolescents, and their parents: a systematic review of the literature. J Psychosoc Oncol.

[27] Otth M, Scheinemann K (2022). Back to school – the teachers’ worries and needs having a childhood cancer patient or survivor in their class. Front Oncol.

[28] Walker AJ, Lewis FM, Lin Y, Zahlis E, Rosenberg AR (2019). Trying to feel normal again: early survivorship for adolescent Cancer survivors. Cancer Nurs.

[29] Cahaney C, Stefancin P, Coulehan K, Parker RI, Preston T, Goldstein J, Hogan L, Duong TQ (2020). Anatomical brain MRI study of pediatric cancer survivors treated with chemotherapy: correlation with behavioral measures, magnetic resonance imaging. Magn Reson Imaging.

[30] Erdmann F, Frederiksen LE, Mogensen H, Pedersen C, Mader L, Talbäck M, Bautz A, Hirvonen E, Kyrönlahti A, Korhonen LM, Hasle H, Malila N, Madanat-Harjuoja LM, Feychting M, Winther JF (2021). Cohort Profile: the socioeconomic consequences in Adult Life after Childhood Cancer in Scandinavia (SALiCCS) Research Programme. Front Oncol.

[31] Godono A, Felicetti F, Conti A, Clari M, Dionisi-Vici M, Gatti F, Ciocan C, Pinto T, Arvat E, Brignardello E, Fagioli F, Pira E (2022). Employment among Childhood Cancer survivors: a systematic review and Meta-analysis. Cancers (Basel).

[32] Brinkman TM, Ness KK, Li Z, Huang IC, Krull KR, Gajjar A, Merchant TE, Klosky JL, Partin RE, Tonning Olsson I, Boop F, Klimo P, Chemaitilly W, Khan RB, Srivastava D, Robison LL, Hudson MM, Armstrong GT (2018). Attainment of Functional and Social Independence in Adult survivors of Pediatric CNS tumors: a Report from the St Jude Lifetime Cohort Study. J Clin Oncol.

[33] Larsen MH, Larsen EH, Ruud E, Mellblom A, Helland S, Lie HC (2022). I have to do things differently now, but I make it work—young childhood cancer survivors’ experiences of self-management in everyday living. J Cancer Surviv.

[34] Papadatou D, Metallinou O, Hatzichristou CLP (2002). Children with chronic and life-limiting conditions: teachers’ perceptions and experiences regarding students’school reintegration. Illn Crisis Loss.

[35] Vanclooster S, Benoot C, Bilsen J, Peremans L, Jansen A (2018). Stakeholders perspectives on communication and collaboration following school reintegration of a seriously ill child: a literature review. Child Youth Care Forum.

[36] Hotchkiss ME, Ahmad ZN, Ford JS (2023). Cancer-peer connection in the context of adolescent and young adult Cancer: a qualitative exploration. J Adolesc Young Adult Oncol.

[37] McDonnell GA, Shuk E, Ford JS. A qualitative study of adolescent and young adult cancer survivors’ perceptions of family and peer support. J Health Psychol. 2018;25(5). 10.1177/1359105318769366. 10.1177/1359105318769366 PMC6167207 29687735

[38] Walker K, Lippard C (2021). Adolescent Cancer survivors’ Oncology Camp Experience. J Child Life: Psychosocial Theory Pract.

[39] Kent EE, Smith AW, Keegan TH, Lynch CF, Wu XC, Hamilton AS, Kato I, Schwartz SM, Harlan LC (2013). Talking about Cancer and meeting peer survivors: Social Information needs of adolescents and young adults diagnosed with Cancer. J Adolesc Young Adult Oncol.

[40] Vandraas KF, Reinertsen KV, Kiserud CE, Lie HC (2021). Fear of cancer recurrence among young adult cancer survivors-exploring long-term contributing factors in a large, population-based cohort. J Cancer Surviv.

[41] Yang Y, Li W, Wen Y, Wang H, Sun H, Liang W, Zhang B, Humphris G (2019). Fear of cancer recurrence in adolescent and young adult cancer survivors: a systematic review of the literature. Psycho-oncology.

[42] Brierley M, Sansom-Daly U, Baenziger J, McGill B, Wakefield C (2019). Impact of physical appearance changes reported by adolescent and young adult cancer survivors: a qualitative analysis. Eur J Cancer Care.

[43] Vuotto SC, Ojha RP, Li C, Kimberg C, Klosky JL, Krull KR, Srivastava DK, Robison LL, Hudson MM, Brinkman TM (2018). The role of body image dissatisfaction in the association between treatment-related scarring or disfigurement and psychological distress in adult survivors of childhood cancer. Psycho Oncol.

[44] Perez GK, Salsman JM, Fladeboe K, Kirchhoff AC, Park ER, Rosenberg AR (2020). Taboo topics in adolescent and young adult oncology: strategies for managing Challenging but important conversations central to adolescent and young adult Cancer survivorship. Am Soc Clin Oncol Educ Book.

[45] Berg T, Böhmer J, Nwaru BI, Karason K, Jarfelt M (2022). Heart failure in childhood cancer survivors-a systematic review protocol. Syst Rev.

[46] Ryder-Burbidge C, Diaz RL, Barr RD, Gupta S, Nathan PC, McKillop SJ, Fidler-Benaoudia MM (2021). The Burden of Late effects and related risk factors in adolescent and young adult Cancer survivors: a scoping review. Cancers (Basel).

[47] Levitch CF, Malkin B, Latella L, Guerry W, Gardner SL, Finlay JL, Sands SA (2021). Long-term neuropsychological outcomes of survivors of young childhood brain tumors treated on the Head Start II protocol. Neurooncol Pract.

[48] Nahata L, Caltabellotta NM, Yeager ND (2018). Fertility perspectives and priorities among male adolescents and young adults in cancer survivorship. Pediatr Blood Cancer.

[49] Wirtz MR, Ahmad ZN, Ford JS (2023). What if I die and no one had ever romantically loved me? Sexual well-being in a sample of YA cancer survivors. J Cancer Surviv.

[50] Levine JM, Kelvin JF, Quinn GP, Gracia CR (2015). Infertility in reproductive-age female cancer survivors. Cancer.

